# Corrigendum

**DOI:** 10.1002/ccr3.6732

**Published:** 2022-12-13

In the article entitled “Cleidocranial dysplasia—A case report of incidentally found and lately diagnosed disorder” that was previously published in Volume 10 Issue 10 of Clinical Case Reports, the surname of seventh author was incorrect.

The correct name should be:

Sobin Pant

We apologize for the error.
